# Spatiotemporal reprogramming of auxin signaling and resistance outputs across model plants and solanaceae crops under *Ralstonia solanacearum* infection

**DOI:** 10.3389/fpls.2026.1820149

**Published:** 2026-06-19

**Authors:** Xiaoxu Li, Xuebo Wang, Xiaobin Wang, Simin Fan, Bingcheng Liu, Xiaonian Yang, Jinhao Sun, Weicai Zhao, Wenxuan Pu, Zhenchen Zhang

**Affiliations:** 1 Technology Center, China Tobacco Hunan Industrial Co., Ltd., Changsha, China; 2 Guangdong Key Laboratory for Crops Genetic Improvement, Crops Research Institute, Guangdong Academy of Agricultural Sciences, Guangzhou, China; 3 Beijing Life Science Academy, Beijing, China; 4 Tobacco Research Institute, Chinese Academy of Agricultural Sciences, Qingdao, China; 5 Tobacco Science Research Institute of Guangdong Province, Shaoguan, China; 6 Guangdong Tobacco Company, Shaoguan, China; 7 Guangdong Provincial Company of China National Tobacco Corporation, Guangzhou, China; 8 Hunan Provincial Key Laboratory of Plant Functional Genomics and Developmental Regulation, College of Biology, Hunan University, Changsha, China; 9 Technology Center, China Tobacco Jiangsu Industrial Co., Ltd., Nanjing, China

**Keywords:** auxin signaling, bacterial wilt, effector-mediated reprogramming, precision resistance, *Ralstonia solanacearum*, root developmental remodeling

## Abstract

Bacterial wilt is a devastating vascular disease caused by the soil-borne bacterium *Ralstonia solanacearum*, which invades and blocks the xylem of susceptible plants, especially economically important solanaceous crops. In addition to classical plant defense hormones, recent research has focused on understanding the role of auxin as a key and continuous regulator of infection and development. Auxin connects changes during infection to root development and the progression of bacterial wilt disease. The auxin related shift in plant hormone patterns associated with root development has been observed in both infected and non-infected host plant species. This shift is accompanied by the upregulation of several auxin regulating transcriptional programs, the accumulation of indole-3-acetic acid, and the enhancement of auxin signaling in specific plant tissues. The auxin-associated reprogramming of roots infected with *R. solanacearum* is also linked to the reorganization of root developmental programs, including reduced primary root elongation, pronounced induction of root hair formation, and increased lateral root development at later stages of infection. Root hair formation in response to infection requires an intact and functional auxin signaling pathway. There is also support for a positive relationship between the level of auxin responsiveness and a plant’s susceptibility to infection. Susceptible genotypes activate their auxin pathway more than resistant genotypes, while reducing the ability to transport or perceive auxin increases plant resistance and decreases the severity of key root-associated phenotypes during infections. At the molecular level, *R. solanacearum* is reported to influence the auxin homeostasis of the host plant indirectly through effectors, which induce changes in the plant immune response, alter metabolism and energy status, and modify the interactions within plant hormone pathways. These changes create conditions that facilitate the colonization and spread of *R. solanacearum*. Therefore, auxin is best understood as a dynamically regulated “balancer” that mediates the trade-off between plant growth and defense, integrating the plant developmental flexibility with immune response. Furthermore, leveraging these findings to develop precision resistance offers strategies for enhancing plant resistance. These include improving auxin modulation within the growth environment and employing spatiotemporal transcriptomics to identify spatially and temporally correlated losses of auxin sensitivity or transport in root zones susceptible to infection.

## 1 Introduction

Bacterial wilt is a devastating vascular disease caused by *Ralstonia solanacearum* that infests a remarkably broad range of over 250 plant species, including important Solanaceae crops such as tomato, potato, pepper, and tobacco (Mansfield et al., 2012; Peeters et al., 2013; Vailleau and Genin, 2023). The pathogen typically invades through wounds in the roots or young root tissues, subsequently colonizing the xylem vessels and leading to systemic wilting and ultimately the death of the entire plant (Peeters et al., 2013; Vailleau and Genin, 2023). As a globally significant plant disease, bacterial wilt has proven notoriously difficult to control. Its extensive host range is partly attributed to the large and diverse repertoire of effector proteins produced by *R. solanacearum* (Landry et al., 2020; Vailleau and Genin, 2023).

These type III secretion system effectors (T3Es) are the pathogen’s primary virulence factors, capable of suppressing plant immune responses while simultaneously hijacking host metabolic processes to benefit the microbe (Coll and Valls, 2013; Landry et al., 2020; Vailleau and Genin, 2023). For a long time, plant defense hormones such as Salicylic acid (SA) and Jasmonic acid (JA) were believed to play dominant roles in resistance against bacterial wilt (Robert-Seilaniantz et al., 2011; Vailleau and Genin, 2023). However, in recent years, accumulating evidence has revealed that auxin also plays a critical role in plant-pathogen interactions, including those involving *R. solanacearum* (Kunkel and Harper, 2018; Kunkel and Johnson, 2021; Kazan and Lyons, 2014). Auxin is a key hormone that regulates plant growth and development and is particularly crucial for root morphogenesis (Overvoorde et al., 2010; Lavenus et al., 2013). An increasing body of evidence suggests that pathogens often manipulate the host’s auxin signaling pathways to enhance their own virulence (Kunkel and Harper, 2018; Kunkel and Johnson, 2021; Kazan and Manners, 2009).

Despite these advancements, several knowledge gaps remain. First, the stage-specific mechanisms by which *R. solanacearum* modulates auxin biosynthesis, transport, and signaling are not fully understood. Second, the molecular determinants that switch auxin from a susceptibility factor to a defense contributor require further elucidation. Third, the spatiotemporal dynamics of auxin distribution within root and vascular tissues during infection have been insufficiently explored. Fourth, most evidence derives from the model plant *Arabidopsis thaliana*, and it remains unclear how directly these findings apply to Solanaceae crops. Finally, the crosstalk between auxin and other immune pathways, including SA, JA, ethylene, and mitogen−activated protein kinase (MAPK), requires systematic integration in the context of bacterial wilt.

In this study, we review recent research advances regarding the role and mechanisms of auxin during the occurrence and development of bacterial wilt, with a focus on how *R. solanacearum* infection alters auxin levels and signaling in the host and how auxin influences the plant’s resistance. We aim to elucidate how auxin pathways are spatiotemporally reprogrammed during infection and to discuss the dynamic balance between growth and defense. This review provides new perspectives for achieving disease resistance through the manipulation of hormonal pathways.

## 2 Effects of *R. solanacearum* infection on host auxin levels and distribution

**Figure 1:**
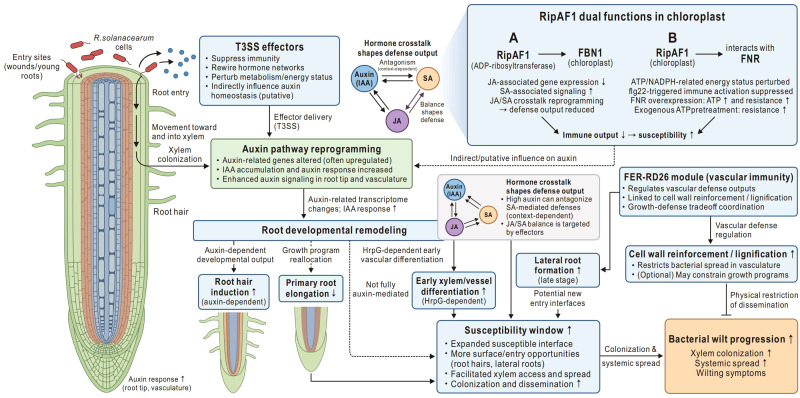
Model of auxin reprogramming and root developmental remodeling during infection.

*R. solanacearum* infection induces a reprogramming of auxin pathways in host roots, characterized by the upregulation of auxin-related genes, accumulation of indole-3-acetic acid (IAA, the primary form of auxin), and enhanced auxin signaling in tissues such as the vasculature (Zhang et al., 2024; French et al., 2018; Zuluaga et al., 2015). This is accompanied by alterations in root development programs. Notably, primary root elongation is inhibited, while root hair formation is dramatically increased, and the number of lateral roots rises (Zhang et al., 2024). Genetic and chemical evidence indicates that some developmental abnormalities, such as induced root hairs, depend on auxin signaling (Lu et al., 2018; Zhang et al., 2024). In contrast, certain changes related to vascular differentiation may not be directly mediated by auxin (Lu et al., 2018). Overall, this coordinated process of enhanced IAA signaling and root structural remodeling likely expands the window of susceptibility in roots and facilitates pathogen entry and spread (**Figure 1**).

### 2.1 Upregulation of auxin-related gene expression and hormone accumulation

A key early host response to *R. solanacearum* infection is the reprogramming of auxin-related gene expression and hormone accumulation. Transcriptomic analyses have shown that in both tomato and wild potato, many auxin-related genes in the roots are significantly upregulated following infection by *R. solanacearum* (French et al., 2018; Zuluaga et al., 2015). In tomato plants, comparisons between resistant and susceptible varieties upon infection reveal significant differences in auxin-related pathways and gene expression responses. The resistant variety tends to suppress certain auxin-responsive genes, whereas the susceptible genotype shows a stronger induction of hormone- and stress-related genes (Zuluaga et al., 2015; French et al., 2018). This suggests that *R. solanacearum* infection generally activates the host’s pathway; however, the specific hormonal response patterns in different hosts may influence the outcome of resistance. At the physiological level, studies further confirm an increase in auxin content within infected tissues. Auxin-reporter analyses and related assays indicate that *R. solanacearum* infection leads to enhanced auxin responses and IAA accumulation in host roots. It has been demonstrated that early xylem vessel differentiation and the precocious formation of xylem elements during infection are dependent on HrpG, a regulator of the pathogen’s virulence. The infection process is accompanied by auxin accumulation and heightened auxin signaling in host roots. Notably, the induction of root hairs by the pathogen requires an intact auxin signaling pathway (Zhang et al., 2024). These results suggest that active infection by *R. solanacearum* can induce a substantial accumulation of auxin in host roots and activate auxin signaling (Lu et al., 2018; Zhang et al., 2024). Notably, changes in the auxin gradient and its distribution often precede the reprogramming of root developmental programs.

### 2.2 Abnormal root development and association with auxin

*R. solanacearum* infection causes multiple abnormal root morphological changes that are closely linked to altered auxin distribution. These include suppression of primary root growth, premature differentiation of root tips, and excessive formation of adventitious roots and root hairs (Lu et al., 2018; Zhang et al., 2024; Yu et al., 2024). In multiple hosts, such as *Arabidopsis*, infection by *R. solanacearum* significantly inhibits primary root elongation and induces the formation of numerous root hairs near the root tip (Lu et al., 2018; Zhang et al., 2024). Zhao et al. (2019) reported that *Arabidopsis* roots exhibit a significant increase in lateral root numbers at later stages of infection. This suggests that while the plant’s early defense responses may suppress lateral root initiation, once the pathogen is established, it manipulates hormone signaling to promote the formation of new lateral roots, thereby providing itself with additional potential entry routes (Kunkel and Harper, 2018; Zhang et al., 2024). Given that auxin is a key regulator of root hair and lateral root formation (Overvoorde et al., 2010; Lavenus et al., 2013), these developmental abnormalities are closely linked to changes in auxin signaling. The study by Lu et al. (2018) was the first to discover that *R. solanacearum* infection can induce abundant root hair differentiation at the host root tip, which is speculated to be related to locally elevated auxin levels (Lu et al., 2018). Subsequent studies confirmed that pathogen-induced root hair formation is indeed dependent on the host’s auxin signaling, as auxin-insensitive mutants exhibited a significantly reduced root hair induction phenotype upon infection (Lu et al., 2018; Zhang et al., 2024). Additionally, *R. solanacearum* infection has been shown to accelerate the differentiation of xylem vessel cells within *Arabidopsis* roots, causing mature vessel elements to appear closer to the root tip than usual (Yu et al., 2024; Zhang et al., 2024). This premature vascular differentiation may represent a strategy by which the bacterium promotes its own spread within the host. However, unlike root hair formation, early xylem maturation is not necessarily directly mediated by auxin, as treatments that block auxin signaling did not abolish this phenotype (Lu et al., 2018; Zhang et al., 2024). In summary, by modulating the host auxin levels and distribution, *R. solanacearum* profoundly alters the normal root developmental program, likely creating physiological conditions more favorable for pathogen invasion and dissemination.

During the infection process of *R. solanacearum*, the distribution of auxin within different regions of the root (the meristematic zone, elongation zone, and differentiation zone) undergoes dynamic changes. In healthy roots, the auxin peak at the root tip maintains meristem activity and prevents premature cell differentiation. However, following infection, the pathogen induces abnormal auxin accumulation in the elongation zone, triggering excessive root hair formation; this phenotype depends on functional auxin signal transduction (Lu et al., 2018; Zhang et al., 2024). In the vascular tissue, changes in the auxin gradient lead to premature differentiation of xylem vessels, which may facilitate bacterial spread through the xylem (Yu et al., 2024; Zhang et al., 2024). Additionally, disruption of polar auxin transport results in auxin accumulation within vascular tissue cells, promoting lateral root germination during the later stages of infection (Zuluaga et al., 2015). These spatial differences indicate that *R. solanacearum* does not uniformly increase auxin levels throughout the root but instead forms localized auxin hotspots, each driving specific developmental abnormalities that collectively promote successful pathogen infection. Emerging spatial transcriptomics techniques are expected to map these auxin gradient patterns at the cellular level with high precision, thereby revealing the specific cell types targeted by the pathogen (Deng et al., 2025).

## 3 Effects of auxin on host resistance to bacterial wilt

Current evidence supports a positive correlation between enhanced auxin signaling and increased susceptibility to bacterial wilt. Susceptible genotypes often exhibit stronger auxin responses upon infection, whereas genetic or chemical attenuation of auxin synthesis, transport, or perception in the host can reduce disease associated phenotypes and improve resistance (French et al., 2018; Lu et al., 2018). However, auxin should not be viewed as a simple “disease-promoting” factor, its role can be a double-edged sword that varies across tissues and stages of infection. The pathogen may exploit auxin to expand the susceptible window, yet it might also participate in the plant’s own processes of damage repair and defense modulation, resulting in a dynamic tug-of-war with immune responses (Kunkel and Johnson, 2021). Therefore, in the context of bacterial wilt, auxin should be regarded as a temporally and spatially fluctuating “balancer”, whose effects depend on growth-defense trade-offs and the strength of its crosstalk with other signaling pathways (**Figure 1**).

### 3.1 Evidence that auxin promotes susceptibility

Elevated auxin levels or signaling are consistently associated with increased host susceptibility in many plant-pathogen interactions, including the *R. solanacearum*-host pathosystem. Multiple lines of evidence indicate that, in many plant-pathogen interactions, elevated auxin levels or signaling are often associated with increased host susceptibility (Kazan and Manners, 2009; Kunkel and Harper, 2018; Kunkel and Johnson, 2021). The *R. solanacearum*-host pathosystem also supports this view. A comparative study of resistant and susceptible tomato varieties found that, during *R. solanacearum* infection, the susceptible variety strongly activated auxin-responsive signaling, whereas the resistant variety tended to suppress the expression of some auxin-related genes (French et al., 2018). This suggests that high levels of auxin signaling may promote pathogen colonization, and that suppression of auxin responses could be a mechanism by which resistant plants limit pathogen invasion (French et al., 2018; Kunkel and Harper, 2018). Supporting this hypothesis, functional evidence comes from genetic studies. Tomato plants carrying the Diageotropica mutation (*dgt1-1*), which disrupts polar auxin transport and causes defects in lateral root development, exhibit enhanced resistance to *R. solanacearum* (French et al., 2018). Similarly, in *Arabidopsis*, the auxin receptor mutant *tir1*, which is less sensitive to auxin, does not develop the extensive root hair phenotype typically observed in wild-type plants upon *R. solanacearum* infection (Lu et al., 2018). Although the *tir1* mutant still shows primary root growth inhibition when infected, the absence of induced root hairs indicates that the auxin signaling pathway is required for this specific pathogen-induced developmental aberration (Lu et al., 2018; Zhang et al., 2024). Moreover, using chemical genetics to inhibit auxin biosynthesis or signaling yields similar conclusions. Treatment with L-kynurenine (L-Kyn), an inhibitor of auxin biosynthesis, significantly reduces the number of root hairs induced by *R. solanacearum* infection, while having relatively little effect on the premature xylem differentiation phenotype (He et al., 2011; Zhang et al., 2024). Taken together, these findings suggest that auxin-mediated morphological changes play an active role in *R. solanacearum* pathogenesis and that excessive auxin signaling likely promotes disease development (Kunkel and Harper, 2018). In general, auxin often “assists” disease progression by promoting abnormal growth in host tissues or by interfering with defense responses (Kazan and Manners, 2009; Kunkel and Johnson, 2021). Notably, an overabundance of auxin signaling can induce the formation of malformed plant tissues and simultaneously suppress salicylic acid (SA)-mediated immune pathways, thereby increasing susceptibility (Robert-Seilaniantz et al., 2011; Kazan and Manners, 2009; Kunkel and Harper, 2018). In *Arabidopsis*, exogenous application of IAA has been shown to enhance the virulence of pathogens (Chen et al., 2007; Mutka et al., 2013; Djami-Tchatchou et al., 2020). Thus, finding ways to appropriately modulate auxin levels is critical for the plant to strike a balance between growth and immunity.

Collectively, genetic, chemical, and comparative evidence strongly supports the conclusion that enhanced auxin signaling promotes susceptibility to *R. solanacearum*. Susceptible genotypes activate auxin pathways more robustly, while impairing auxin synthesis, transport, or perception consistently reduces disease severity and root abnormalities. Although the precise molecular mechanisms remain to be fully elucidated, the trend across multiple hosts and experimental approaches is clear: auxin primarily functions as a susceptibility factor in bacterial wilt.

### 3.2 Auxin and the balance of resistance

Despite auxin’s predominant role in promoting susceptibility, it does not always act unilaterally; in certain contexts, auxin signaling can also contribute to defense. It is important to note that auxin does not always act unilaterally to promote susceptibility. In certain contexts, activation of auxin signaling may also contribute to the plant defense response, albeit through complex mechanisms (Kunkel and Johnson, 2021). Some studies have proposed that the increase in auxin observed during *R. solanacearum* infection might produce a “double-edged sword” effect in local tissues: on one hand, elevated auxin levels can benefit the pathogen by inducing host developmental aberrations that compromise structural integrity; on the other hand, the plant may be attempting to use auxin signaling to activate defense-related genes or to accelerate the repair of damaged tissue (Kazan and Manners, 2009; Kunkel and Harper, 2018). Mechanistically, whether auxin signaling favors susceptibility or contributes to defense appears to depend on its spatiotemporal dynamics, which crosstalk with SA and other hormone pathways, and whether its downstream outputs are biased toward developmental reprogramming or toward tissue repair and vascular immune reinforcement. Notably, a recent study has further expanded our understanding of auxin biodynamics by highlighting the integral roles in enhancing plant response to biotic and abiotic stresses, which proved that auxin functions as a central hub integrating growth and defense signaling (Ali et al., 2025b). Currently, the precise mechanisms by which auxin exerts both positive and negative effects on disease resistance remain unclear and require further investigation.

Notably, Kunkel and colleagues have reported that auxin functions not only as a plant hormone influencing host resistance but also as a signal detected by pathogens, directly affecting the expression of specific bacterial genes and behaviors (Kunkel and Harper, 2018; Djami-Tchatchou et al., 2020). Overall, substantial evidence indicates that elevated auxin levels generally increase susceptibility to *R. solanacearum*. However, the dynamic balance between auxin and resistance is context-dependent, varying over time and space, and is modulated through crosstalk with other signaling pathways. A more detailed analysis of this balance is necessary to fully understand how auxin simultaneously regulates growth and defense.

## 4 Molecular mechanisms of pathogen manipulation of host auxin

*R. solanacearum* likely manipulates the host’s auxin pathway indirectly and through multiple steps. Its effector proteins can suppress immunity, disrupt host metabolism and energy status, and reshape hormonal networks, thereby driving changes in the host’s auxin homeostasis (Vailleau and Genin, 2023; Wang et al., 2025). Although direct evidence of an effector targeting core auxin signaling components is still lacking for this pathogen, the bacterium may alter auxin distribution by affecting upstream processes such as auxin biosynthesis or transport in the host. Ultimately, the interplay of auxin with SA, jasmonic acid (JA)/ethylene, and vascular immunity modules like the FER-RD26 pathway collectively shapes disease progression and resistance outcomes (Kong et al., 2020; Wang et al., 2024).

### 4.1 Effector protein-mediated signal interference

*R. solanacearum* manipulates host auxin pathways indirectly through a suite of effector proteins that reprogram host cell physiology, suppress immunity, and alter metabolism. The bacterium relies on its effector proteins to reprogram host cell physiology, thereby indirectly manipulating endogenous hormones such as auxin (Landry et al., 2020; Vailleau and Genin, 2023). To date, no *R. solanacearum* effector has been reported to directly interact with auxin receptors or core auxin transcription factors; however, studies on other pathogens provide analogous insights. For example, the type III effector AvrRpt2 from *Pseudomonas syringae* promotes the degradation of host Aux/IAA repressor proteins, thereby accelerating auxin signal transduction and increasing host susceptibility (Chen et al., 2007; Cui et al., 2013). Similarly, some fungal pathogens secrete auxin analogues or induce the host to produce auxin to facilitate their colonization (Han and Kahmann, 2019; Kazan and Lyons, 2014). In *R. solanacearum*, multiple effectors have been shown to disrupt plant hormone pathways and metabolic processes in ways that favor infection (Landry et al., 2020; Vailleau and Genin, 2023). One effector, RipAF1, was recently found to significantly impact the host’s hormone network and immune response. RipAF1 possesses ADP-ribosyltransferase activity and can directly modify the host fibrillin protein FBN1. Transient expression of RipAF1 in plants suppresses the expression of JA-related genes while inducing SA-associated signaling. Importantly, its ADP-ribosylation enzymatic activity is required for modulating the antagonistic crosstalk between the JA and SA pathways (Wu et al., 2024). In addition to interfering with hormone signaling, RipAF1 can disrupt the host’s chloroplastic energy metabolism. Wang et al. (2025) demonstrated that RipAF1 interacts with the host enzyme ferredoxin: NADP^+^ reductase (FNR) in the chloroplast; FNR is involved in the generation of NADPH and ATP. Overexpression of FNR alone in plant cells increased ATP accumulation and enhanced resistance to *R. solanacearum*, but co-expression of RipAF1 significantly reduced ATP levels. Furthermore, pre-treating plants with exogenous ATP bolstered their resistance to *R. solanacearum* infection (Wang et al., 2025). This suggests that *R. solanacearum* effectors like RipAF1 may induce a “bottom-up” energy deprivation in host cells, weakening plant defenses and indirectly affecting numerous processes, including hormone biosynthesis and transport. In summary, the diverse functions of *R. solanacearum* effectors enable the bacterium to manipulate the host on multiple levels: directly suppressing immune signaling while also creating an internal environment favorable to the pathogen by disrupting hormone balances and metabolic pathways (Landry et al., 2020; Vailleau and Genin, 2023). Although no effector has yet been identified that specifically targets auxin signaling, it remains possible that some uncharacterized effectors influence auxin biosynthesis or transport, thereby triggering the observed auxin accumulation and root developmental disorders. In other pathogens, such as certain symbiotic rhizobia, effectors have been found to affect the stability of auxin transporters, thus altering the host’s hormone distribution (Kazan and Lyons, 2014; Kunkel and Johnson, 2021). In summary, although none *R. solanacearum* effector has been found to directly target core auxin signaling components, cumulative evidence indicates that effectors such as RipAF1 indirectly promote auxin accumulation and altered distribution by suppressing SA defenses, depleting host energy, and disrupting hormone crosstalk. The absence of a direct effector-auxin interaction does not diminish the importance of auxin reprogramming; rather, it suggests that the pathogen employs a multi-pronged, indirect strategy to induce auxin dysregulation.

### 4.2 Potential contribution of pathogen-derived auxin

An additional possibility is that *R. solanacearum* itself may synthesize and secrete IAA to directly manipulate the host, though direct evidence is currently lacking. Beyond hijacking host pathways, whether *R. solanacearum* itself can synthesize and secrete IAA to manipulate the host is an important question. Many plant pathogens possess tryptophan metabolic pathways and can produce IAA as a signaling molecule (Kunkel and Johnson, 2021; Aragón et al., 2014; Cerboneschi et al., 2016). For instance, in *Pseudomonas syringae* pv. tomato DC3000, enzyme involved in IAA metabolism and regulation, such as the IAA-lysine synthetase encoded by the *iaaL* gene, contributes to the bacterium’s survival and virulence inside plants (Djami-Tchatchou et al., 2022, Djami-Tchatchou et al., 2020). In the case of *R. solanacearum*, genomic analyses have not definitively identified a canonical IAA biosynthesis cluster; however, considering the pathogen’s large genome and metabolic flexibility, the existence of an unconventional IAA production pathway cannot be ruled out. Even more intriguing is the fact that auxin influences not only plant cells but also bacterial behavior: studies have shown that exogenous IAA can regulate gene expression in certain pathogens and affect their quorum sensing and virulence traits (Kunkel and Harper, 2018; Djami-Tchatchou et al., 2020). Therefore, if *R. solanacearum* is capable of synthesizing or inducing the production of IAA, the auxin could act bidirectionally, affecting plant cell physiology while simultaneously signaling bacterial cells, thereby achieving a form of reciprocal regulation. At present, there is no direct evidence that *R. solanacearum* produces and secretes IAA to promote infection, but this hypothesis merits further experimental investigation.

### 4.3 Auxin interactions with other hormones

The outcome of bacterial wilt is shaped by complex crosstalk between auxin and other defense hormones, particularly SA, JA, and ethylene. The plant hormone network is highly complex, with auxin interacting extensively with other defense hormones. During the progression of bacterial wilt, such hormonal crosstalk significantly influences disease development. It is well known that auxin and SA signaling often act antagonistically: high auxin levels tend to suppress SA-mediated resistance, while SA accumulation can inhibit auxin signaling (Robert-Seilaniantz et al., 2011; Kazan and Manners, 2009; Kong et al., 2020). *R. solanacearum*, a broad-host-range pathogen, triggers defense responses characteristic of both biotrophic and necrotrophic interactions, resulting in a complex hormone response profile in the plant (Robert-Seilaniantz et al., 2011; Vailleau and Genin, 2023). Studies have shown that during the initial phase of *R. solanacearum* infection, the host does not strongly activate JA signaling; instead, some SA- and ethylene-related defense genes are upregulated (Vailleau and Genin, 2023). This may represent a specific defense strategy employed by the plant against a vascular pathogen. However, the subsequent upregulation of auxin could undermine the SA pathway, creating a negative feedback loop that benefits the pathogen. Recent research indicates that the activation of certain structural defenses can also trigger changes in auxin signaling. When plants deposit large amounts of lignin to reinforce the cell wall, auxin-mediated cell elongation is inhibited, thereby slowing growth (Yu et al., 2024). In the context of bacterial wilt, cell wall fortification is a crucial resistance mechanism that can impede bacterial spread within the xylem (Macho, 2016; Xue et al., 2025; Vailleau and Genin, 2023). However, excessive cell wall defense can come at the expense of normal plant growth. The FER-RD26 module has been identified as a regulator of vascular immunity that influences defense responses related to the cell wall and lignification (Wang et al., 2024). This feedback mechanism exemplifies how plants strive to balance “growth” and “resistance”. It is postulated that the FER-RD26 module helps coordinate this trade-off by modulating downstream signals, thereby fine-tuning the degree of cell wall strengthening versus growth inhibition. Although the detailed mechanisms remain to be elucidated, it is clear that plant resistance to *R. solanacearum* is not governed by a single hormone; rather, it results from multiple interwoven signals, with auxin serving as an important component in this complex signaling network. Although less studied, auxin signaling may also crosstalk with MAPK cascades. Several *R. solanacearum* effectors are known to target MAPK pathways, which could indirectly modulate auxin homeostasis (Asai et al., 2002; Meng and Zhang, 2013). Emerging evidence indicates that reactive species, particularly reactive oxygen species (ROS), play a critical regulatory role in plant defense. ROS can modulate auxin signaling by affecting the stability of PIN proteins and the redox state of auxin receptors (Ali et al., 2025a). This crosstalk may be especially relevant during *R. solanacearum* infection, as several bacterial effectors are known to directly regulate the host ROS burst (Ju et al., 2025).

### 4.4 Transcription factors mediated signal interference

Recent studies have demonstrated that the outcome of auxin signaling (enhancing susceptibility or promoting defense) depends on the specific Aux/IAA inhibitory factor combined with the ARF transcription factor involved, as well as the cellular environment. For example, in *Arabidopsis*, IAA7/AXR2 and IAA3/SHY2 participate in pathogen-induced root hair formation (Lu et al., 2018). Homologous proteins ARF7, ARF19, and several IAA genes are present in tomato and potato, and their expression changes following infection by *Xanthomonas campestris* pv. solanacearum (French et al., 2018). In tomato, the dageotropica (*dgt*) mutant carries a mutation in a holin protein gene that regulates auxin transport and signaling, exhibiting enhanced resistance accompanied by increased lignin deposition and altered auxin-responsive gene expression (French et al., 2018; Rivera-Zuluaga et al., 2023). The DGT holin protein can interact with transcription factors such as SlbZIP1, SlbZIP29, and SlMYB110, suggesting a regulatory link between auxin signaling and transcriptional reprogramming related to defense (Rivera-Zuluaga et al., 2023). Comparative transcriptomic analyses indicate that disease-resistant tomato varieties suppress the expression of auxin-related genes during infection, whereas susceptible varieties strongly induce these genes (French et al., 2018). In peppers, multiple WRKY transcription factors, including CaWRKY3, CaWRKY6, CaWRKY22, CaWRKY27, CaWRKY30, and CaWRKY40, positively regulate the immune response to *Xanthomonas campestris* pv. solanacearum, and their expression is modulated by salicylic acid and ethylene signaling pathways (Hussain et al., 2021, Hussain et al., 2024). Although these WRKY factors are not direct components of the auxin signaling pathway, their interaction with auxin signaling may influence the growth-defense balance. In potatoes, genome-wide analyses have identified 27 Aux/IAA and 21 ARF family members, many of which respond to stress treatments (Kumar et al., 2015; Wu et al., 2017). Functional validation of these transcription factors in solanaceous crops is necessary to clarify their specific roles in resistance or susceptibility. Identifying homologous regulatory modules in solanaceous crops remains a key focus for future research.

## 5 Implications and outlook: from “hormone homeostasis” to “precision resistance”

Given auxin’s significant role in the pathogenic mechanism of *R. solanacearum*, future strategies for managing bacterial wilt could explore regulating hormonal balance as a novel approach. For instance, developing screening methods for varieties with reduced sensitivity to auxin signaling may be beneficial. For example, the tomato *dgt1–1* mutant, which exhibits defective auxin transport and impaired lateral root formation, shows resistance to *R. solanacearum* (French et al., 2018). This suggests that molecular breeding aimed at reducing root sensitivity to auxin or diminishing auxin transport capacity could potentially produce new varieties with enhanced resistance to bacterial wilt. However, it is important to note that such modifications may cause growth defects, necessitating careful fine-tuning to avoid yield penalties. From the perspective of balancing growth and defense, restricting these modifications to specific tissues or developmental stages is crucial. This could potentially be achieved by employing pathogen-inducible expression systems that confer strong resistance only during infection, without adversely affecting the plant under normal conditions. Alternatively, chemical regulation offers a flexible approach. Recently, Liu et al. (2023) conducted small-molecule screening and identified several compounds that inhibit the receptor-like kinase FERONIA (FER). This inhibition enhanced root immunity and protected plants against *R. solanacearum* infection. These compounds function by relieving the negative regulation imposed by FER on cell wall reinforcement, thereby boosting resistance, a strategy that exemplifies “promoting defense by inhibition” of a suppressor. Similarly, it is worth considering whether “auxin antagonists” could be safely applied to crops to suppress auxin signaling during infection. Currently, some synthetic auxin analogues or antagonists are used in research to modulate plant development; if it can be demonstrated that they improve disease resistance without significantly impairing plant growth, they could be potential candidates for disease control (Hayashi et al., 2012; Hasegawa et al., 2018). In addition to these approaches, emerging omics technologies will significantly enhance our understanding of the dynamic hormonal changes during disease progression. The rapidly advancing field of spatiotemporal transcriptomics, for example, enables researchers to resolve gene expression patterns at the tissue or even cell-type level (Deng et al., 2025). Applying this technology to the plant-*R. solanacearum* interaction could produce a detailed map of auxin-related gene expression across different root zones throughout infection, helping to identify key tissues or cell types with distinct hormone responses (Deng et al., 2025). This is crucial for determining whether pathogen-induced auxin signaling is a localized or systemic event. Indeed, single-cell and spatial transcriptomic analyses have begun to demonstrate their power in studies of plant development and pathology, although challenges remain in data interpretation and cross-validation across tissue contexts (Deng et al., 2025). In future studies, integrated transcriptomic, proteomic, and metabolomic analyses should be combined to systematically uncover the regulatory networks linking auxin signaling with immune responses during *R. solanacearum* infection, and to clarify how pathogen effectors reshape these interconnected pathways.

Screening germplasm for natural variation in auxin-related genes could identify alleles that confer enhanced resistance without growth penalties. Genome-wide association studies (GWAS) in tobacco and potato have identified quantitative trait loci (QTLs) associated with bacterial wilt resistance; however, few of these QTLs have been linked to auxin-related genes (Lai et al., 2021; Okiro et al., 2025). This suggests that either auxin signaling components are not major contributors to natural resistance variation or that they have been underexplored in GWAS panels. To address this gap, future research should screen natural populations of Solanaceae crops for allelic variants in auxin biosynthesis genes, auxin transport genes, and auxin signaling genes, and assess their correlation with resistance to *R. solanacearum*. Identifying such variants could provide breeders with molecular markers to select bacterial wilt-resistant varieties without compromising normal root development.

Although much of our current mechanistic understanding is derived from model plants such as *Arabidopsis*, the conservation of core auxin signaling components across Solanaceae suggests that these principles are broadly applicable (French et al., 2018). However, direct validation in crop species is essential. To address this, we propose leveraging recent breakthroughs in spatial transcriptomics and single-cell RNA sequencing (scRNA-seq), which are now being applied to plant stress responses (Yu et al., 2025). These technologies can generate high-resolution maps of auxin-related gene expression across specific root zones and cell types in Solanaceae crops during infection. This approach would clarify whether the pathogen-induced auxin response is a local or systemic event, directly addressing the spatiotemporal dynamics emphasized in this review (Deng et al., 2025). Ultimately, such precision resistance strategies will be key to uncoupling the detrimental effects of auxin on defense from its essential roles in growth.

## 6 Conclusion

In conclusion, current evidence supports a dynamic model in which *R. solanacearum* manipulates host auxin biology across infection stages. Early infection is associated with auxin-related transcriptional activation and accumulation, intermediate stages with root developmental remodeling, and later stages with the maintenance of susceptibility through broader effector-mediated rewiring of hormone crosstalk and host metabolism. On one hand, the pathogen induces auxin accumulation and developmental reprogramming in the host to facilitate infection; on the other hand, the plant simultaneously attempts to utilize or constrain auxin signaling to balance growth and defense. In summary, auxin has become an indispensable factor to consider in the study of *R. solanacearum* pathogenesis. Looking ahead, a deeper understanding of how *R. solanacearum* manipulates host auxin at the molecular level will provide new targets for developing resistance strategies. Disrupting the critical interactions between pathogen effectors and the host auxin pathway, or fine-tuning the host’s hormonal equilibrium, could enhance plant resistance to bacterial wilt. At the same time, these lines of inquiry hold significant theoretical importance for understanding how plant root development and immune responses are balanced. In the face of global crop security challenges, leveraging molecular genetics, chemical biology, and advanced omics tools to modify hormone signaling pathways may emerge as an innovative approach for controlling persistent diseases like bacterial wilt.
